# Supplementary material for: Patient Activation, Depressive Symptoms, and Self-Rated Health: Care Management Intervention Effects among High-Need, Medically Complex Adults
Source: Int J Environ Res Public Health. 2021 May 26;18(11):5690. doi: 10.3390/ijerph18115690 (PMC8198245; doi:10.3390/ijerph18115690)
Supplement: Supplementary file 1 [file ijerph-18-05690-s001.zip › ijerph-1222898-supplementary.pdf]

Chronic Care Management Implementation: Exemplar Case

Mary May is 62 years old and is a patient at the Greene Street Clinic. Mary has multiple chronic conditions including diabetes, hypertension and high cholesterol. Last year she had a foot amputated due to diabetes mismanagement. Though Mary seems to want better health, she doesn't know where to start. She has visited the emergency department several times in recent months due to high blood sugar readings and she is afraid of losing her mobility and her independence. Mary's initial Patient Activation Measure (PAM) score was at level one. At the end of the one-year chronic care management intervention, Mary's PAM score progressed to a level three.

The comprehensive assessment completed by the social worker (SW) provided a baseline of how Mary related to her environment, what supports she had and areas where she may need assistance from community resources. For example, Mary did not have a functioning blood glucose meter or an adequate supply of test strips. The SW assisted her to obtain these supplies and set up auto-delivery to ensure adequate test strip supplies in the future. Using the PAM results, the registered nurse (RN) engaged in relationship building and assisted Mary to set initial goals for her health action plan. Based on those goals, Mary to learned how to use her blood glucose meter and observe how she felt when her glucose was at different levels. The RN coached Mary on the use of her meter, having Mary demonstrate the skill and problem solve basic barriers. Eventually, Mary was able to make the connection between her health and her blood sugar levels. The RN also obtained a list of medications Mary was taking every day and then conducted a comprehensive medication review in collaboration with the Greene Street Clinic pharmacist. Discrepancies between the medications Mary was taking and those that were ordered by the prescribing health professionals were resolved. The RN coached Mary to set up a medication schedule and obtained a pill box to help her better track her medication-taking.

In a subsequent visit approximately 7 months after Mary's enrollment in the study, the RN reviewed Mary's most recent PAM scores. It became evident that Mary has begun to realize her role in her health and how her behavior impacts the way she feels as evidenced by her progression to PAM level two. At this stage, setting very achievable goals to build confidence was of key importance. The Health Action Plan was reviewed and updated. Mary elected to set a nutritional goal of replacing one snack per day with a fruit or vegetable. With coaching and reflection, she began to understand how nutritional choices affect her blood glucose levels and how she feels. The team continued to work with Mary to build her knowledge and understanding of her conditions, as well as her ability to actively participate in her health. The Care Manager assisted Mary in role playing out her next appointment with her provider at the Greene Street Clinic. The role play helped Mary decrease her anxiety and she develop a written list of questions to ask her provider during the visit. The RN and SW also helped Mary develop a plan about using Urgent Care instead of going to the ER if Mary becomes concerned about her diabetes, but it does not appear to be life threatening. Further, they began working on basic problem-solving skills and introduced healthy stress management activities, such as breathing and relaxation techniques.

Once Mary achieved some small successes and recognized her role as an active participant in her health, she began setting some larger goals. At 9 months, the PAM confirmed that Mary had achieved level three. The RN and SW continued to support and encourage her in problem solving and goal setting. Mary set a goal to begin regular physical activity. The SW helped her locate a water therapy/aerobic program at the local YMCA and obtained a reduced-price membership and a transportation plan. While Mary remained in PAM level three at the end of the study, she had the potential to achieve level four because she was making healthy eating, physical activity and diabetes management a routine priority in her life. She had has also become an active participant of her health care team, acknowledging her role in her health and the possibility for positive health in the future.
